# Aberrant One-Carbon Metabolism and Ancestral Genetics Underlie Edematous Severe Acute Malnutrition

**DOI:** 10.21203/rs.3.rs-6890799/v1

**Published:** 2025-06-29

**Authors:** Neil Hanchard, Natasha Lie, Yixing Han, Qing Li, Aarti Jajoo, Jared Redmond, Aparna Haldipur, Solome Zewdu, Emilyn Banfield, Shanker Swaminathan, Sharon Howell, Orgen Brown, Roa Sadat, Nancy Hall, Katharina Schulze, Thaddaeus May, Marvin Reid, Mark Manary, Indi Trehan, Mamana Mbiyavanga, Wisdom Akurugu, Colin McKenzie, Dhriti Sengupta, Elizabeth Atkinson, Ananyo Choudhury, Kwesi Marshall, Carolyn Taylor-Bryan

**Affiliations:** National Human Genome Research Institute; Baylor College of Medicine; National Institutes of Health; National Human Genome Research Institute; Harvard Medical School; National Human Genome Research Institute; National Human Genome Research Institute; National Human Genome Research Institute; National Human Genome Research Institute; Baylor College of Medicine; University of the West Indies; University of the West Indies; Baylor College of Medicine; Baylor College of Medicine; Baylor College Of Medicine; Baylor College of Medicine; University of the West Indies; Washington University at St. Louis; University of Cape Town; University of Cape Town; University of the West Indies; University of the Witwatersrand; Baylor College of Medicine; University of the Witwatersrand; University of the West Indies; University of the West Indies

**Keywords:** kwashiorkor, marasmus, genomics, 1-carbon metabolism, admixture, local ancestry

## Abstract

Severe acute malnutrition (SAM) contributes to the death of millions of children under age five annually. SAM is clinically classified as non-edematous SAM (NESAM) or the more severe edematous SAM (ESAM), which is more common in east-central Africa and the Caribbean. The reason some children develop ESAM while others develop NESAM remains unclear; however, recent studies have identified aberrant one-carbon metabolism (OCM) in ESAM relative to NESAM. Here, we assess genetic variants at 103 loci known to influence OCM, and determine their association with ESAM in 711 samples from Jamaica and Malawi. Seven OCM loci showed evidence of association across both populations, including five associated with homocysteine and folate metabolism (*MTHFR*, *AHCYL1*, *PRICKLE2*, *GABBR2*, and *PLD2*). Three SNPs in *PLD2*, *PRICKLE2*, and *GABBR2*, genotyped using cell-free DNA from serum metabolomic samples, supported causal effects on ESAM risk through homocysteine-related metabolites. Cumulatively, OCM-related variants showed more association with ESAM than expected by chance (z = 3.06), with differing effect magnitudes in the two populations. By leveraging chromosomelevel patterns of intracontinental African admixture, we demonstrate that OCM variant associations with ESAM occur on a shared east-African ancestral genetic background. Finally, using whole genome sequence data from eight African populations, we demonstrate that several OCM loci have outlier signatures of selection in multiple populations, including the ESAM-associated *PLD2* locus. These findings strengthen support for aberrant OCM in ESAM pathogenesis, with implications for current interventions, and highlight the potential of cell-free DNA, intra-continental admixture, and population genetics in mapping disease risk.

## INTRODUCTION

Severe acute malnutrition (SAM) affects 16.9 million children worldwide, predominantly those under five years old, and either directly or indirectly contributes to more than one million childhood deaths per year^1,2^. SAM is clinically characterized by a weight-for-height that is more than three standard deviations below the median, a mid-upper-arm circumference of less than 115 mm, or the presence of nutritional edema^3^. In practice, SAM is recognized to occur in two clinically distinct forms: edematous SAM (ESAM), which includes the syndromes of kwashiorkor and marasmic-kwashiorkor, and non-edematous SAM (NESAM), also known as marasmus^4^. ESAM accounts for a higher proportion of hospitalized SAM in east- and central- Africa and the Caribbean^5,6^, and is associated with mortality rates of 10–20% among those who are hospitalized with SAM and medical complications^7-9^. ESAM is clinically characterized by bilateral pitting edema (swelling) of the extremities and severe systemic involvement that may include skin and hair changes, fatty liver, and multi-system organ failure^4,10,11^. By contrast, NESAM is more prevalent in Southeast Asia and northern- and western-African geographies, and is characterized by generalized wasting of body tissues, typically with less severe systemic involvement^4,6^. Whilst it is well-recognized that the development of SAM is largely linked to food security and availability, the persistence of adverse geopolitical factors mean that SAM is still highly prevalent globally.

ESAM was first described in medical literature in the early part of the 20th century^12-14^; however, the reasons why a severely malnourished child will develop ESAM as opposed to NESAM remain largely unknown^15^. In the past, it was hypothesized that diet or protein intake were primary contributors to ESAM risk; however, decades of epidemiological surveys have failed to show consistent evidence for differences in protein intake or any other single dietary deprivation between children who develop ESAM and those who develop NESAM^16-19^. Similarly, there have been no consistently demonstrated differences in infectious or environmental exposures between children who develop ESAM and those who develop NESAM^4,20-22^. There are, however, numerous pathophysiological differences between the two clinical manifestations, particularly in their metabolism and biochemistry; for example, ESAM has been associated with a slower rate of protein breakdown in starvation, impaired heparin sulfate proteoglycan expression, greater dietary cysteine efficiency, and increased oxidative stress, compared to children with NESAM^23-28^. Despite this, it remains unknown whether these observations represent downstream effects that result from the disorder or are primary etiological differences. Perhaps as a result, ESAM and NESAM are still treated in the same manner, using the same nutritional supplements, despite well described differences in pathophysiology and underlying biochemistry.

Among biochemical differences between children with ESAM and those with NESAM, the cellular movement of methyl groups known as one-carbon metabolism (OCM) has been a particular recent focus. OCM plays a major role in health and disease, providing substrates for mitochondrial activity and ATP generation, dNTPs for DNA repair, and methyl groups needed for the maintenance of DNA and other cellular methylation events in mitotically active cells^29^. The broad metabolic reach of OCM has been hypothesized as a potential unifying factor for the multisystem dysfunction seen in ESAM, and recent studies lend support to this. For instance, metabolomic studies have demonstrated that children with ESAM have significantly lower serum levels of the essential amino acid methionine^30^ with concomitant related effects, including dysregulation of asymmetric dimethyl arginine (ADMA), lower cysteine levels, impaired cysteine synthesis, reduced acylcarnitine production, and impaired fatty acid transport^25,31^. Mice fed a maize-vegetable diet similar to that seen among SAM children in Malawi, develop a fatty liver phenotype that is similar to that seen in ESAM, which is abrogated by supplementation of the methyl-group donor choline^32^. Similarly, although there are no overt differences in microbiome composition between NESAM and ESAM children, a metagenomic study of severe malnutrition, conducted without regard to subtype, reported significantly lower OCM metabolite levels among gnotobiotic mice transplanted with stool from children with ESAM^33^.

Observations of OCM dysfunction in ESAM are also consistent with stable isotope whole-body flux studies reporting slower methionine flux among children with ESAM^26^. We have previously shown that during the acute nutritional stress, children with ESAM have significantly lower buccal cell DNA methylation than children with NESAM^34^, and this was consistent with observations of methionine turnover. Notably, and consistent with turnover studies conducted in children who had recovered from SAM, methylation differences were not observed among adults who had recovered from having either ESAM or NESAM as children, confirming that OCM dysfunction is a feature of the acute nutritional insult. Significantly hypomethylated loci were associated with genes both directly and indirectly implicated in the ESAM phenotype, and with loci associated with disordered nutrition states (e.g., obesity) and body anthropometry. Thus, collectively, there is a growing body of evidence supporting OCM dysfunction in ESAM. These findings, however, do not directly explain why only some children have the OCM derangements associated with ESAM, whilst others from the same region and environment have the OCM profile associated with NESAM.

**Figure 1:**
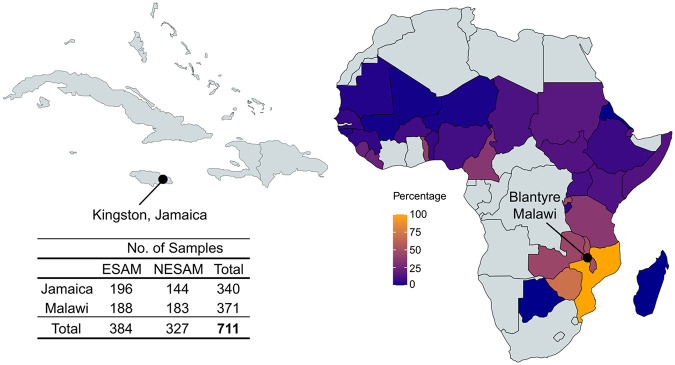
SAM samples and study recruitment in Jamaica and Malawi are shown alongside the prevalence (%) of nutritional edema (ESAM) across Africa from “Putting Kwashiorkor on the Map”^7^.

Although there are no formal heritability estimates for ESAM, children with repeated bouts of SAM are more likely to develop the same form of SAM (i.e., either ESAM or NESAM) upon readmission to hospital^35,36^; this observation, alongside the well-documented lack of demonstrable environmental, infectious, or metagenomic differences between ESAM and NESAM, has focused attention on the potential genetic risk of ESAM, which is consistent with recent interest in the genetics of malnutrition^37^. To date, however, attempts at genetic association have been limited to three single-candidate SNP association studies conducted in sample sizes of less than 200 individuals decades ago^38-40^. We thus sought to take a modern genetic approach to understanding the risk of developing ESAM, leveraging advances that have characterized genetic risk association studies of other public health disorders, including nutritional disorders like obesity. OCM is a fundamental metabolic pathway that has been genetically characterized^41,42^, with well-curated genes and loci mediating interindividual metabolic variation. Therefore, we started by evaluating the potential impact of genetic variation at OCM loci on the risk of developing ESAM. In this study, we compile a comprehensive list of OCM-associated genetic loci and evaluate genetic association with ESAM at these loci in population samples from Jamaica and Malawi (Fig. 1). We then leverage population genetics and both local and global population ancestry, to further explore the association between OCM and ESAM.

## MATERIALS AND METHODS

### Study participants

Details of participant samples used in the study have been previously published^34,43^. All recruitment consisted of samples collected after individuals had received a diagnosis of either ESAM or NESAM.

Samples from Jamaica included DNA from blood and buccal samples from children (< 18 years) diagnosed with SAM. DNA was extracted using a phenol/chloroform protocol as described previously^43^. SAM subtype was determined using the Wellcome Classification^44^, where NESAM (marasmus) is defined as < 60% weight-for-age without edema, and ESAM includes marasmic-kwashiorkor- defined as < 60% weight-for-age with edema, and kwashiorkor - defined as 60–80% weight-for-age with edema. Pediatric participants were recruited prospectively from the Tropical Metabolism Research Unit of the Caribbean Institute for Health Research at the University Hospital of the West Indies (UHWI), the major tertiary nutritional center on the island. Recruitment was part of a larger, long-running study of the genetic etiology of SAM. A cohort of adult participants (> 18 years) who formerly had SAM as children were recruited from the same site. The study was approved by the Ethics Committees of the UHWI/University of the West Indies Faculty of Medical Sciences.

In Malawi, samples from children with SAM were recruited from 18 rural sites across five districts^34^. Samples were originally collected between 2013–2016. The sub-type of SAM was defined using mid-upper arm circumference (MUAC) and the presence or absence of edema. Buccal samples were collected using the Oragene Discover (OGR-250) DNA collection kit (DNA Genotek Inc., Ottawa, Ontario, Canada). The protocol was modified to collect buccal epithelial cells by swabbing the inside of each cheek ten times. DNA was extracted following the manufacturer's instructions. After collection, to maintain clinical uniformity in SAM designation between Jamaica and Malawi, samples from Malawi were re-classified using the Wellcome Classification as having either ESAM or NESAM, which was highly concordant with the MUAC based designation. The study was approved by the National Health Science Review Committee of the Ministry of Health, Government of Malawi.

Written informed consent was obtained from the parents or adult guardians of children included in this study. Permission to use de-identified participant samples for genetic studies at Baylor College of Medicine (BCM) was approved by the Institutional Review Board (IRB) of BCM and confirmed by the National Institutes of Health IRB subsequently.

### Curation of OCM loci

GeneWeaver^45^, AmiGO 2^46,47^, GWAS Catalog^48,49^, KEGG^50^, NCBI BioSystems^51^, Pathway Commons^52^, and PubMed were queried using the search terms “one-carbon metabolism”, “cysteine”, “methionine”, and “one-carbon pool by folate” to identify OCM loci to be used in this study. At the genome level, a 50 kilobase (kb) buffer region was included on either side of each identified gene to capture potential *cis*-acting elements. This search resulted in a list of 103 genes involved in OCM. Overlapping regions were consolidated into single regions, resulting in 95 loci (**Supplementary Table S1**).

### Genotyping and quality control

Peripheral blood and buccal DNA samples from Jamaican and Malawian individuals were genotyped for this study. Downstream quality control and analyses are shown in **Supplementary Figure S1**. Genotyping was performed on the Infinium H3Africa Consortium Array v1.1 (web resources), which provides LD coverage of ~ 0.89 in the African superpopulation of 1000 Genomes. Raw idat files were processed in GenomeStudio (Illumina, San Diego, California, USA), including within-sample clustering, to create binary input files for PLINK. Genotype fluorescence projections in GenomeStudio were visually inspected for those SNPs that surpassed test-wide significance in order to confirm genotype calls. Downstream SNP and sample quality control (QC) was performed in PLINK v1.07^53^ and included limiting to biallelic autosomal SNPs and excluding SNPs with a missingness > 0.95; removal of SNPs outside the Hardy-Weinberg test distribution; and excluding samples with missingness > 10%. Linkage disequilibrium (LD) pruning was conducted using a window size of 50 bases, a sliding window of ten bases, and r^2^ threshold of 0.1. Pairs of samples with a proportion-of- identity-by-descent (IBD) (PI_HAT) score > 0.1 were flagged, and the individual within the pair with the higher missingness rate was removed. Post QC, 1.8 million genotyped SNPs were available for downstream analysis across the 711 remaining samples.

### Imputation

Given the relative lack of representation of African genomes in existing imputation panels, we chose to conduct a two-stage imputation and data merge process. Data from Jamaican individuals (n = 340) were imputed using the Consortium on Asthma among African-ancestry Populations in the Americas (CAAPA) reference panel^54^, which was generated using data from individuals with predominantly west-African ancestry, including Jamaicans. We used minimac3 v1.0.5 (University of Michigan, Ann Arbor, Michigan, USA)^55^ and Eagle v2.3 (Broad Institute, Cambridge, Massachusetts, USA)^56^ on the Michigan Imputation Server (University of Michigan, Ann Arbor, Michigan, USA)^55^ to perform phasing and imputation on our Jamaican samples, selecting the African reference panel ("AFR") as the reference population.

Data from Malawian participants (n = 371) was imputed using the H3Africa reference panel (H3ABioNet)^57^ (web resources), which is designed to represent a wide array of African ancestries, including east- and central-African groups that neighbor Malawi. Phasing was performed using Eagle 2.4^56^ (Broad Institute, Cambridge, MA, USA). Minimac4 Version 1.0.0 (University of Michigan, Ann Arbor, MI, USA) was used for imputation on the H3ABioNet Nextflow chip imputation pipeline^58^, which implements a similar workflow to the Michigan Imputation Service pipeline, and was deployed on the University of Cape Town High-Performance Computing Facility (web resources).

After imputation, SNPs with r^2^ < 0.3 were excluded. The imputed data from both population groups was merged using an in-house command line script (web resources), prior to removing non-autosomal SNPs and SNPs with minor allele frequency (MAF) < 0.05. The final imputed dataset included 8,415,377 SNPs, of which 45,411 SNPs were at OCM loci (+/−50kb of genes).

### Multidimensional scaling

Genome-wide SNPs were used to perform multidimensional scaling (MDS) in PLINK v1.07, and these were used for two different purposes (see **Supplementary Figure S1**): 1. - on the QC-ed genotype dataset used for association analyses; 2. on the genotype-only dataset alongside datasets from 1000 Genomes^59^ to put the primary populations in the context of African datasets (See Population ancestry and admixture mapping below). 1000 Genomes datasets consisted of Esan individuals in Nigeria (ESN, n = 99), individuals from Gambia in Western Division, the Gambia (GWD, n = 113), Luhya individuals in Webuye, Kenya (LWK, n = 99), Mende individuals in Sierra Leone (MSL, n = 85), and Yoruba individuals in Ibadan, Nigeria (YRI, n = 108). The genotyped-only dataset was merged with the above 1000 Genomes populations, and LD-pruned with a window size of 50 bases, a window of 10 bases, and r^2^ < 0.1. MDS was performed PLINK and visualized using an in-house R^60^ script (**Supplementary Figure S2**).

### Genetic association analyses

Association statistics were primarily derived using Genome-Wide Efficient Mixed Model Association (GEMMA)^61^, which better accounts for genetic ancestry variation. Mixed model association analysis in GEMMA was performed using the -lm 4 flag. Standardized relatedness of the combined genotyped and imputed dataset was also calculated using the flag -gk 2. We used the genetic data to estimate the standardized kinship matrix before applying the generalized linear mixed effect model to assess genetic association between SNPs and the risk of developing ESAM, assuming random effect on individuals and fixed effects due to sex and the first component of MDS (as suggested by scree plots of the proportion of variance explained; **Supplementary Figure S3**). In the regional regression analyses, the MDS component was estimated from the dataset of the specified cohort. QQ plots were generated using the R package qqman^62^. Manhattan plots were generated using an in-house script (web resources).

### Cumulative association

We employed a permutation test procedure to assess the cumulative association between OCM loci and SAM status; this is a non-parametric method of assessing the statistical significance of observed data without making assumptions about the underlying probability distribution of the data. We hypothesized that OCM loci are enriched for SNPs with p-values below a certain significance threshold, i.e., the proportion of significant loci within OCM loci is more than expected by chance. We choose p = 0.001 as a representative significance threshold for the number of loci being tested (similar to a Bonferroni correction for multiple testing of 0.1/100). To test the robustness of our findings, we included a ceiling threshold of p = 0.01, and two equidistance points (0.001 and 0.01) as sensitivity points.

The test procedure involved taking the genome-wide association statistics for all genotyped SNPs (n = 1,843,153) and calculating the proportion of SNPs with a p-value meeting a pre-specified threshold. We then randomly sampled a subset of SNPs equivalent to the number of genotyped SNPs across OCM loci (n = 9,479) and determined the proportion of those SNPs with a p-value meeting the predefined threshold. That step was repeated 10,000 times to derive an empirical distribution of the proportion of significant SNPs. We then calculated a z-score for the observed proportion based on the empirical distribution. To account for the randomness of genome-wide SNPs relative to a restricted metabolic pathway, we performed the same test using a similar number of SNPs sampled from loci involved in ketone metabolism.

### Haplotype association and calculation of linkage disequilibrium

**Figure 2:**
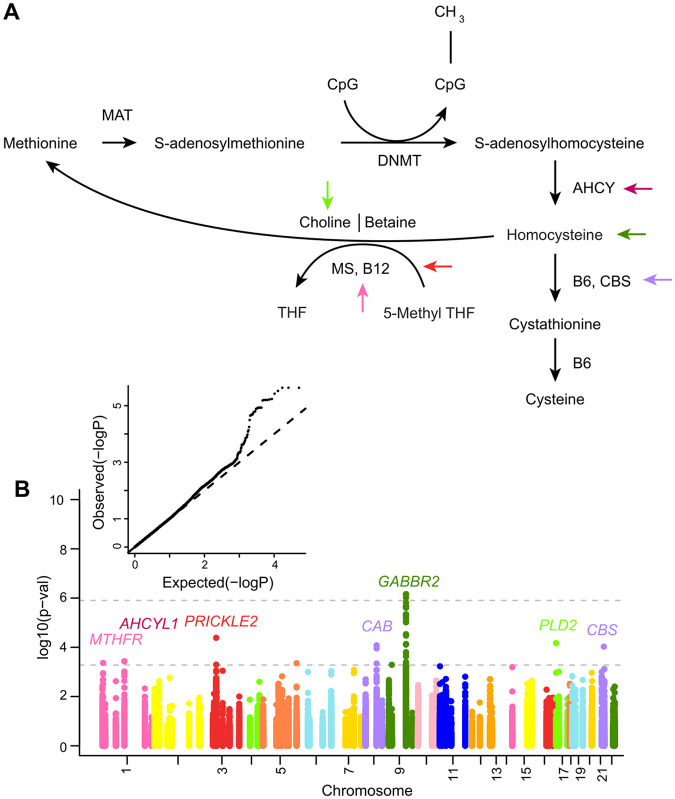
**A** - The one-carbon metabolism (OCM) cycle showing the folate and methionine cycles. In the folate cycle, dietary folate is processed to 5-methyl-tetrahydrofolate, which is then used in the production of methionine from homocysteine. Methionine is processed in its eponymous cycle to produce homocysteine, which contributes methyl groups for DNA and other cellular methylation and can be remethylated to methionine through the folate cycle. **2B** - QQ plot and Manhattan plot of SNP (genotyped and imputed) association with ESAM demonstrating candidate loci. The activity of loci reaching the locus threshold for significance (dashed line at –−logP= 3.27) or the SNP threshold for significance (dashed line at −logP= 5.9) in OCM is represented by colored arrows in 2A matching the locus colors in 2B.

Genotyped SNPs in *GABBR2* (n = 5) and *PRICKLE2* (n = 6) reaching region-level significance (Fig. 2) were arranged in 5' to 3' order and phased in PLINK v1.07 to create basic region-level haplotypes. PLINK was also used to determine association using the --hap-assoc flag. PLINK files were also parsed to Haploview^63^ in order to generate plots of linkage disequilibrium.

### Population ancestry and admixture mapping

To investigate the ancestral African genetic ancestries in the Jamaica and Malawi datasets, genome-wide genotype data was merged with selected population data from publicly available datasets using PLINK v1.9 (**Supplementary Figure S1**). Available datasets included the African Genome Variation Project (AGVP)^64^, H3Africa Consortium^65^, Schlebusch *et al.*^66^, and 1000 Genomes Project (Phase III). Only SNPs common to all datasets were retained. As genetic reference data is not available for every African ethnolinguistic group, we sought to ensure that we could assess contributions from Niger-Congo speaking groups, which includes Bantu speakers - the largest ethnolinguistic grouping across the continent, as well as non-Niger-Congo groups, such Rain Forest Forager, Hunter-Gatherer (e.g. Khoe and San), and Nilo-Saharan groups, which are more populous in east- and central- Africa. Populations representing Niger-Congo African ancestry included Bantu speakers from Zambia (BSZ, n = 39), Jola (n = 79), and YRI (n = 100). Populations representing non-Niger-Congo African ancestry included Amhara (n = 42), Botswana (BOT, n = 48), LWK (n = 74), Khoe and San Hunter Gatherers (n = 18), Zulu (n = 95), and MSL (n = 85). Samples from CEU (n = 95) were included to represent European admixture. The merged dataset was further filtered to remove SNPs with excessive missingness (> 0.1) and then LD-pruned (see MDS above). The final merged genotype dataset included ~ 50,000 autosomal SNPs. The resulting merged dataset was used to perform MDS for a subset of groups using the same parameters and procedures as above.

Using the unsupervised clustering algorithm as implemented by ADMIXTURE v1.3^67^, global ancestry proportions were inferred for all populations. Since the algorithm is known to be impacted by the number of individuals that represent a population, 100 random individuals each from Jamaica and Malawi were retained to approximate the sample sizes of the reference populations. ADMIXTURE was performed for K = 2 to K = 6, and the K with the lowest cross-validation error estimates was noted (K = 5).

### Local ancestry inference

**Supplementary Figure S1B** illustrates the data processes for local ancestry. Data subset from 1000 Genomes consisting of CEU, MSL, and LWK individuals was used as references for European, West African, and East African ancestry, respectively. The SAM cohort dataset and the 1000 Genomes dataset each underwent independent QC filtering using MAF > 0.05; SNP missingness threshold of 0.05; and removal of duplicate SNPs. The remaining SNPs were merged using an in-house script. The merged dataset was subject to additional QC filtering to remove A > T and G > C SNPs. Datasets were then phased by chromosome using ShapeIt2^68^, using the 1000 Genomes Phase 3 genetic map with default parameters.

Local ancestry inference scores were calculated for the genotyped-only dataset using RFMix version 2^69^. Local ancestry for the reference 1000 Genomes dataset was run separately from the SAM cohort dataset to maximize the available data generated on differing platforms, and 1000 Genomes-specific indices were used as reference ancestry groups. The 1000 Genomes Phase 3 genetic map was used for RFMix, which was otherwise run with default parameters.

### Ancestry-adjusted association analyses

In ancestry-adjusted association tests, we employed TRACTOR v1.1.0^70^. SNP association with ESAM disease risk was performed whilst accounting for the ancestral origin of the SNP by first assigning ancestry labels to the two haplotypes of individuals, referred as tracts. Tracts were extracted from the genotyped-only dataset using ExtractTracts.py from TRACTOR, with integers from one to three referring to the three proxy ancestries - CEU, LWK, and MSL - from 1000 Genomes. RunTractor.py was then run to perform logistic regression using NESAM vs ESAM status as the outcome for each individual, with tracts, ancestry-specific allele counts, and sex as regressors. OCM loci were then subset from the regression results and visualized^62^.

### Cell-free DNA genotyping of serum metabolomics samples

Cell-free DNA (cfDNA) was obtained from serum samples of 415 individuals from Malawi. These individuals were independently recruited as part of our published metabolomics study of one-carbon metabolites in SAM^31^. Samples were initially stored at −80°C and subsequently slowly brought up to room temperature to minimize degradation. CfDNA was extracted using the Qiagen QIAamp MinElute Virus Spin Kit (Cat #57704), which is optimized for cfDNA isolation, and yielded a mean concentration of 2.7ng/μL of genomic DNA per sample, with concentrations ranging from 0.1 ng/μL to 52.0 ng/μL.

We attempted to amplify six ESAM-associated SNPs from cfDNA samples – two in *PRICKLE2* (rs753562 and rs17664202), two in *GABBR2* (rs17664203 and rs17664204) and one each in *SHMT1* (rs651495) and *PLD2* (rs1052748). We were unable to obtain consistent or robust genotype data for rs651495 (*SHMT1*) and rs17664203 (*GABBR2*) and results from these assays were not included in downstream analyses. Primers for the remaining SNPs were designed using an *in silico* primer design tool (https://www.ncbi.nlm.nih.gov/tools/primer-blast/) using the SNP ID (rsID) in GRCh38/hg38 (**Supplementary Table S2**). Primer specificity was confirmed using BLAT. Primers were synthesized by IDT (Coralville, IA, USA) and prepared as 100 μM stock solutions in TE buffer. PCR amplification was performed using 4ng of DNA under a single thermocycling and amplification protocol (**Supplementary Methods**), with products verified via gel electrophoresis (GelDoc, BioRad laboratories, Hercules, CA, USA). Following PCR, products were purified using Exo-Sap IT (Thermo Fisher Scientific, Waltham MA, USA) to remove excess primers and dNTPs. Dideoxy- (Sanger) sequencing was conducted using M13-tagged primers and the Big Dye v3.1 sequencing kit (Thermo Fisher Scientific, Waltham MA, USA). Sequencing reactions were cleaned using the Qiagen DyeEx kit (Qiagen, Germantown, MD, USA) to remove dye terminators, followed by genotype calling on the SeqStudio (Genetic Analyzer #A33770, Fisher Scientific, Waltham MA, USA). Sequencher (software version 4.10.1) was used to visualize and confirm genotype calls.

### Mendelian randomization (MR) using serum metabolites

Following the same classification criterion used in our primary cohort, we selected 252 samples with diagnoses of ESAM (n = 135) and NESAM (n = 117) for whom we had cfDNA genotypes *and* metabolomic data for all 16 OCM metabolites - choline, betaine, dimethylglycine (DMG), glycine, sarcosine, 5-methyltetrahydrofolate (MTHF), serine, methionine, S-adenosylmethionine (SAMe), S-adenosylhomocysteine (SAH), homocysteine, cysteine, cystathionine, pyridoxal phosphate (PLP) and asymmetric dimethylglycine (ADMA). The remaining samples were either community controls or had a diagnosis of moderate acute malnutrition.

Mendelian randomization (MR) was used to assess the causal effect of individual metabolites using the genotypes as the instrument variables. For this analysis, we performed two-sample MR based on multiple SNPs by combining results from the SNP-metabolite and the SNP-outcome associations to show potential *in vivo* effects. We applied one instance of the inverse-variance approach (IVW) from MendelianRandomization R package. Under this approach, the causal effect is obtained from a weighted linear regression of the associations with the outcome, on the associations with the metabolite, while fixing the intercept to zero and weights being the inverse-variances of the associations with the outcome^71^. We also exploited the MR-Egger method to detect invalid instrument variables and bias^72^. The serum metabolites were highly correlated (R2). Our cumulative association data suggested that there might be synergistic effects among SNPs; therefore, we assumed an additive genetic model for test alleles, and then tested for the causal effect on ESAM risk of each metabolite, after log10 transformation (to better approximate normality). We selected three genotyped SNPs as instrument variables, one SNP from each locus, with SNP rs753562 in gene *PRICKLE2* being omitted because the serum genotype data was slightly out of Hardy-Weinberg equilibrium and the SNP is physically close to the other *PRICKLE2* SNP. The estimates of SNP-outcome association are drawn from the GEMMA analysis of the Malawi group in the primary cohort with fixed effects being sex and the first component of MDS. The estimates of SNP-metabolite association are drawn from a linear regression of log_10_(metabolite) with sex and age as covariates.

### Whole genome sequencing

A subset of 151 DNA samples from Malawi were chosen to undergo whole-genome sequencing to assess recent positive selection in OCM-related genomic regions. These samples were selected to have an approximately equal distribution of males and females and an equal number of ESAM and NESAM samples to minimize sampling bias. WGS was performed by first generating PCR-free libraries from one microgram genomic DNA using the TruSeq^®^ DNA PCR-Free HT Sample Preparation Kit (Illumina, San Diego, CA, USA). The median insert sizes were approximately 400 bp. Libraries were tagged with unique dual-index DNA barcodes to allow the pooling of libraries and to minimize the impact of barcode hopping. Libraries were then pooled for sequencing on the NovaSeq 6000 (Illumina) to obtain at least 300 million 151-base read pairs per individual library. The sequence data were then aligned to human_g1k_v37_decoy.fasta via BWA-MEMv0.7.15^73^. GATK Resource Bundle B37^74^ was used to sort the data, mark duplicates, perform BQSR, and run the GATK Haplotype Caller to generate gVCF files on each sample. Then individual gVCFs were merged into a multi-sample VCF file using GATK 4.3.0.0. GATK Best Practice^75^ for variant calling and filtration was followed to obtain high-quality variant data, i.e., applying GATK VQSR function, followed by harder filters (filter parameters set as DP > = 10, QUAL > = 60, and ExcessHet < = 54.69).

### Signatures of selection

We used the iHS score^76^ to identify genomic signatures of selection in the dataset; this analysis is agnostic of phenotype and covariates. To compare signatures of selection at OCM loci between different countries, we created subsets of country-specific signatures of selection generated by the H3Africa consortium^65^, which included selection statistics for SNPs across the OCM loci used in the association study: Benin (n = 45,537 SNP scores), Botswana (BOT, n = 44,277 SNP scores), Cameroon (CAM, n = 44,458 SNP scores), Mali (MAL, n = 43,033 SNP scores), Berom from Nigeria (BRN, n = 44,177 SNP scores), Gur speakers from West Africa (GWR, n = 43,523 SNP scores), and Zambia (ZAM, n = 47,572 SNP scores). These samples were sequenced on Illumina HiSeq 2500 and subsequently processed and mapped to GRCh37. Integrated haplotype scores (iHS) were calculated using selscan^77^. We calculated iHS scores for the Malawi WGS dataset using the same QC and run parameters as the H3Africa datasets. SNPs with iHS scores > ∣2∣ were considered outliers, potentially under selection. Selection signatures were visualized using R^60^ and ggplot2^78^.

## RESULTS

Our primary analyses utilized DNA samples from 833 individuals from Jamaica and Malawi approximately evenly split between ESAM (case/affected group) and NESAM (reference/control group) at each location (Fig. 1; **Supplementary Table S3**). Samples were genotyped on the Illumina H3Africa array, with subsequent quality control (QC) processing resulting in 711 samples (**Supplementary Figure S1**) and 1,639,325 genotyped SNPs (Methods). Among genotyped SNPs, 9,479 SNPs fell within 10kb (+/−) of a curated list of 103 autosomal genes and loci previously associated with OCM (Methods; **Supplementary Table S4**); this included four gene clusters with two or more genes in tandem. After variant imputation and quality control (Methods), a set of 45,411 imputed and 9,479 genotyped SNPs at 95 OCM loci were identified to test for association and perform downstream analyses (**Supplementary Figure S1**).

### Variation at OCM loci is associated with ESAM

We first assessed the association between imputed SNPs at OCM loci and ESAM using the linear mixed model implemented in GEMMA (see Methods). The top associated SNP (rs79824961 near *GABBR2*) surpassed our multiple-testing adjustment for the total number of SNPs interrogated (p < 9.73x10^−7^) (Fig. 2). A further 56 SNPs at nine OCM loci, surpassed a secondary significance threshold adjusted for the number of loci (n = 95) tested (p < 5.26x10^−4^). This latter threshold was supported by an excess of observed p-values without evidence of test-wide inflation (λ = 1.02) on the resulting QQ plot (Fig. 2). To further reduce potential false positives, we characterized candidate loci as those with multiple SNPs surpassing our locus-level cut-off or single variants with evidence of association using a secondary method (n = 3 loci; Methods). This resulted in a final set of seven ESAM-associated OCM loci (**Supplementary Table S4**).

The strongest associated locus was an intragenic region on chromosome 9q22.33 (**Supplementary Figure S4A**) falling within the first intron of gamma-aminobutyric acid type B receptor subunit two (*GABBR2*; top SNP rs7038285, p = 6.98x10^−7^, Odds Ratio (OR) = 0.85), where the minor allele (C) was enriched among individuals with NESAM. SNPs within intron 13 of *GABBR2* (~260kb upstream of our top SNPs) have been associated with lower plasma homocysteine^41^ in a European population. The mechanistic relationship of this association to the *GABBR2* gene, which is a member of the G-protein coupled receptor 3- and GABA-B receptor families, are unclear. We also found similar evidence of putative association at *PRICKLE2* on chromosome 3p14.1 (top SNP rs11130959, p = 4.15x10^−5^, OR = 0.88) **(Supplementary Figure S4B**), with the minor allele also being enriched among NESAM participants. The canonical function of *PRICKLE2* is unknown, although it has been associated with variation in folate levels^79^. Of the seven top candidate loci, five (*MTHFR*, *AHCYL1*, *PRICKLE2*, *GABBR2*, and *PLD2*) directly impact either conversion to- or breakdown of- homocysteine (Fig. 2), including a coding variant rs1052748 (p.Thr577Ile) in exon 17 of *PLD2* that was enriched among ESAM individuals **(Supplementary Figure S4C**). *PLD2* encodes phospholipase D2 phosphatidyl, which generates the methyl-group donor choline through the hydrolysis of phosphatidylcholine. The rs1052748 variant is reported as an expression quantitative trait locus (eQTL) for *PLD2* in multiple tissues in the Gene-Tissue Expression database (GTEx)^80^.

**Figure 3:**
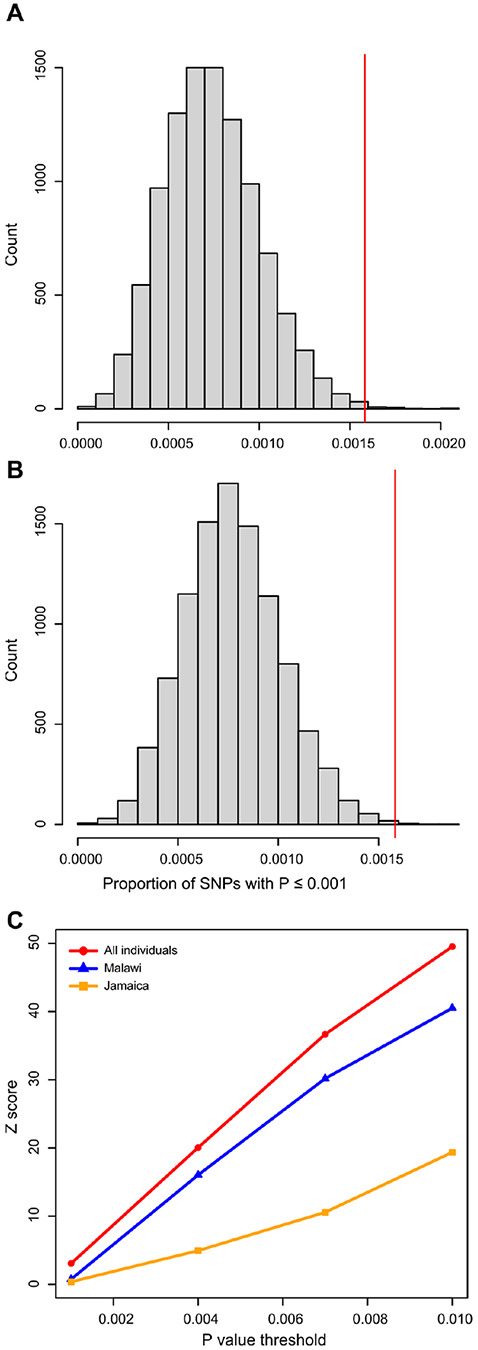
Histogram illustrating the proportion of 10,000 randomly sampled SNPs surpassing a significance threshold of p< 0.001, compared to the number of SNPs in OCM loci passing the same threshold (red line). **3A** - SNPs were randomly sampled from the total genotyped pool. **3B** - SNPs randomly sampled from a pool of ketone metabolism loci. **3C** - Z scores of random sampling experiments for p-value thresholds between locus-level significance threshold and a p value ceiling of p=0.01 (see methods).

Metabolic flux results from cumulative enzymatic activity at multiple points within a biochemical cycle. Given our observation of multiple putatively associated OCM SNPs, each with variable effects on OCM, we considered the potential for a cumulative effect of OCM-associated SNPs on ESAM susceptibility. To test this, we applied a permutation test (Methods) to the association statistics of OCM SNPs and compare this with the association tests of the ~ 1.8 million genotyped SNPs genome wide (for which no SNP surpassed genome-wide significance). At a p-value cut-off of < 0.001, a greater proportion of SNPs at OCM loci were associated with ESAM relative to the randomly sampled dataset ((z = 3.2; Fig. 3a). At more permissive p-value thresholds, the z-score differentiating our OCM SNPs and the random sampling experiment was even more pronounced (e.g., at a threshold of p = 0.01, the resulting z-score was 50 (Fig. 3c)).

Next, we considered whether our cumulative association might have resulted from comparing SNPs in linkage disequilibrium (LD) at metabolically related loci to a random distribution of largely unlinked SNPs. To do so, we repeated the permutation test, but this time randomly sampling the same number of SNPs (n = 9,479) from the 51,105 SNPs found at loci related to ketone metabolism, which is not known to be associated with SAM (Methods). Relative to this ketone metabolism background, the proportion of OCM SNPs showing association was still in the upper tail of the distribution (z = 3.19) at the p < 0.001 threshold (Fig. 3b). Finally, as a negative control, we performed the same genome-wide comparison as above for SNPs at sphingolipid metabolism loci, which has a similar number of SNPs to OCM, but no *a priori* evidence for involvement in ESAM. The proportion of sphingolipid metabolism SNPs showing association with ESAM fell well within the randomly generated distribution (z= −0.70).

There are few established models for evaluating genetic variation in SAM. Therefore, we sought to provide support for the proposed functional impact of associated variants by assessing the impact of ESAM-associated SNPs on OCM metabolites in the context of ESAM vs NESAM. We first extracted cell-free DNA (cfDNA) from ~ 400 serum samples (Methods) from our previous study of OCM metabolites in ESAM/NESAM, which was done in an independent cohort of children from Malawi recruited around the time of their SAM diagnosis^31^. We then genotyped four ESAM-associated SNPs (rs753562 (*PRICKLE2*), rs17664202 (*PRICKLE2)*, rs17664204 (*GABBR2*), and rs651495 (*PLD2*) using cfDNA samples (Methods; **Supplementary Methods**). Three SNPs (rs17664202 was out of Hardy-Weinberg equilibrium) were then used as instrumental variables in a two-sample Mendelian Randomization (MR) to assess the causal effect of each of the 16 OCM metabolites on ESAM risk (Methods).

**Figure 4:**
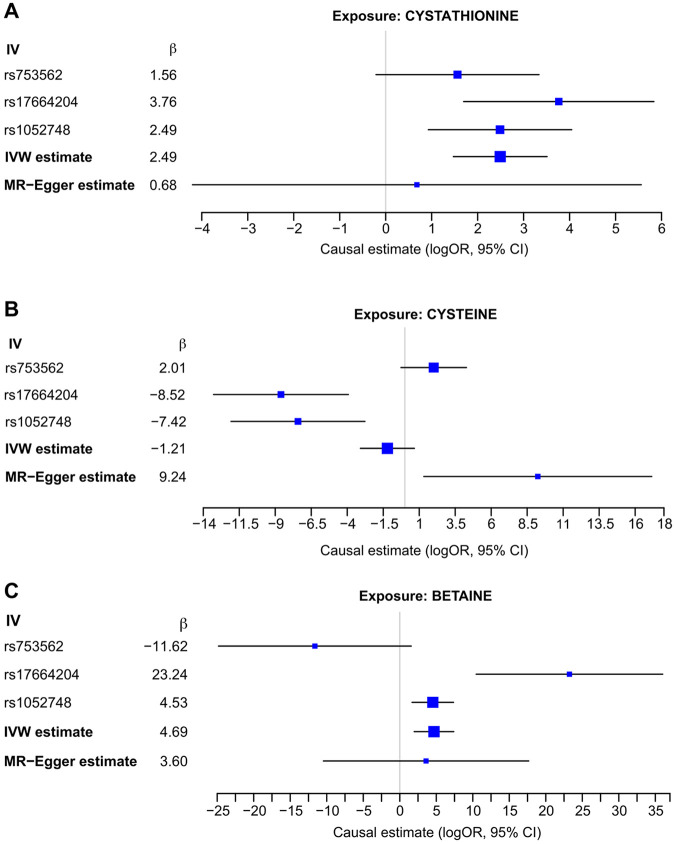
Causal effects estimated using a Two-Sample Mendelian Randomization (MR) approach to modelling combined effects from three independent SNPs. Estimates of SNP-exposure association are drawn from analyses of serum metabolites adjusting for sex and age; the estimates of SNP-disease association are drawn from GEMMA analyses of the Malawi group in the primary cohort. **4A** – Forest plot of causal SNP effects on cystathionine in the risk of developing ESAM vs NESAM. **4B** – Forest plot of causal SNP effects of cysteine; using MR-Egger, the log-odds ratio is estimated at 9.24 (p = 0.02). **4C** – Forest plot of causal SNP effects on betaine. Using the IVW approach, the estimated log odds ratio is 4.69 (p=0.0007), although Cochran's Q test p<0.001. IV – Instrumental Variable; CI – Confience Interval; OR – Odds Ratio.

We first assessed SNP-metabolite and SNP-disease associations separately under regression models and then combined effects using the IVW method assuming additive genetic effects. Although OCM metabolites are highly correlated, we found significant casual effects on cystathionine (log-odds ratio ~ 2.49, p = 1.65x10^−6^) and on betaine (log-odds ratio ~ 4.69, p = 0.0007) (Fig. 4). With three SNPs, there was substantial genotypic heterogeneity in the causal effect of betaine and cysteine (p < 0.001 by Cochran's Q test for both). We thus reevaluated the model after excluding any SNPs with opposing effects (although such effects could reflect the effect of the major allele), which necessarily limited our analyses to the IVW approach. Using the two SNPs with congruent effects, the causal effect of cysteine was estimated ~ −7.97, (p = 2.24x10^−6^ and Cochran’s Q test p = 0.74) and the estimate for betaine was 5.41, (p = 0.0001 and Cochran’s Q test p = 0.005). Although we did not find strong evidence for cystathionine or betaine under the MR-Egger approach, we found that estimates from both approaches were similar in direction and magnitude.

### Population ancestry influences the association of OCM loci with ESAM

In the absence of an available secondary cohort in which to replicate our findings, we also sought evidence of internal consistency in the magnitude and direction of effect between the two countries represented in our cohort. At our seven candidate loci, MAFs and directions of effect among associated SNPs were similar between the two countries (**Supplementary Table S5**). Despite similarities in the direction of effect in the two countries, we noted that the magnitude of the effect was generally stronger in Malawi than Jamaica despite comparable sample sizes and minor allele frequencies (**Supplementary Figure S5**). This difference was also evident when we looked at statistically inferred haplotypes (Methods) comprised of associated (genotyped) SNPs at our two top loci – *GABBR2* and *PRICKLE2*. We observed evidence of association with ESAM in each population group; however, the haplotypes with the strongest implied effect differed between the two population groups despite similar patterns of LD in the two groups at both loci (**Supplementary Figure S6**). Similarly, when we stratified our cumulative association analyses by population group, there was always a higher proportion of association SNPs in Malawi (Fig. 3c).

**Figure 5:**
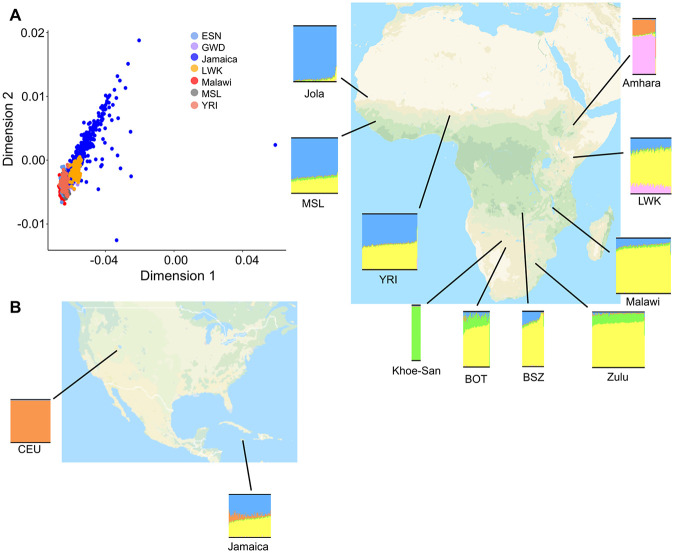
Admixture and Ancestry in the SAM cohort. **5A** – Multidimensional scaling dimensions (C1 vs C2) for Jamaican and Malawian SAM cohort in the context of African (AFR) populations from 1000 genomes (ESN – Esan in Nigeria; GWD – Gambians from Western Division; LWK – Luyha in Webuye, Kenya; MSL - Mende in Sierra Leone; YRI – Yoruba in Ibadan, Nigeria). **5B** - ADMIXTURE plots (K=5) for Jamaicans and Malawians in the SAM cohort shown in geographical context of putative ancestral source populations (CEU – Utah Residents (CEPH) with Northern and Western European ancestry; BOT – Botswana residents; BSZ – Bantu Speakers from Zambia from the H3Africa Consortium).

To explore this further, we first considered our populations in multidimensional scaling (MDS) space. Jamaican and Malawian samples clustered with other African continental populations on MDS components one and two, although samples from Malawi were tightly clustered alongside other Bantu-speaking Niger Congo ethnolinguistic groups (YRI, MSL). Conversely, Jamaican samples were more dispersed (Fig. 5a; **Supplementary Figure S2**), likely a result of the diverse population ancestry origins of Jamaicans, which include a predominant contribution of African ancestry from the British slave trade of the 14th to 17th centuries, as well as a subsequent influx of indentured peoples from East- and Southeast- Asia, and Europe, in addition to centuries of European (British and Spanish) colonialization.

We then used ADMIXTURE^67^ to infer patterns of genome-wide admixture in our cohort in the context of parental proxies of geographic and continental differentiation derived from the African Genome Variation Project (AGVP)^64^, the Human Health and Heredity in Africa (H3Africa) Project^65^, and 1000 Genomes (1KG) Project^59^ (Methods). In this analysis, we proxied European ancestry through the Ceph from Utah (CEU); West African ancestries through the Yoruba from Nigeria (YRI) and Mende from Sierra Leone (MSL); East African ancestries through the Luhya from Webuye, Kenya, and the Amhara from Ethiopia; and southern African ancestries through individuals from Botswana (BOT), Bantu-speakers from Zambia (BSZ), the Zulu, and Khoe and San Hunter-Gatherer groups (KS). As expected, samples from Jamaica had, on average, a significantly higher proportion of European (~ 13%) and west African ancestry (~ 68%) than Malawians, in whom east-African ancestries (~ 83%) were more common (Welch two-sample t-test, p = 2.2x10^−16^) (Fig. 5b; **Supplementary Figure S7**).

### Association between OCM loci and ESAM is driven by shared east-African ancestry

Given the ancestral genetic differences between the two study populations, concomitant differences in associated haplotype backgrounds, and reported geographical variation in ESAM prevalence, we considered that the underlying OCM-ESAM association might be best represented by an ancestral haplotype that is shared by the two populations but seen more commonly in Malawi. In this shared ancestry model, the presence of additional ancestry backgrounds (admixture) might obscure the underlying association signal, especially if using genome-averaged ancestry to account for population stratification. To evaluate this, we used RFmix^69^ to derive ‘local’ (locus-level) patterns of ancestry, using the CEU, MSL, LWK populations to proxy European, West African-, and East African- Bantu-speaking parental ancestries, respectively, in our cohort (Methods). We then repeated our OCM-wide association tests, but this time adjusting each SNP for its ancestral haplotype background using TRACTOR. This approach allowed us to account for the potential effects of differing ancestral haplotype backgrounds at putatively associated loci in a way that was agnostic to geography but still ancestrally sensitive.

**Figure 6:**
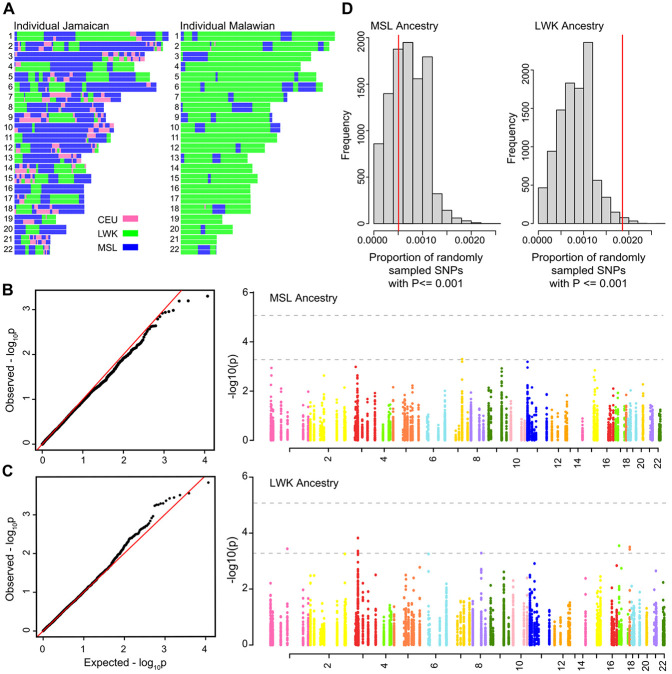
Local ancestry association. **6A** - illustrative local ancestry plots for Jamaican and Malawian individuals. **6B** – QQ plot and Manhattan plot for TRACTOR-based association conditioning on West African (MSL) ancestry. **6C** - QQ plot and Manhattan plot for TRACTOR-based association, conditioned on East African (LWK) ancestry. **6D** – Cumulative association (see methods) by ancestry group showing the empirical distributions of the proportion of 5,916 SNP associations in TRACTOR with P<0.001 after resampling 10,000 times. The red line shows the observed proportions for 5,916 SNPs at OCM loci.

Consistent with genome-wide admixture estimates, Jamaican individuals harbored more blocks of European- and west-African- derived ancestry than Malawi (Fig. 6a). Across the combined cohort, however, the overall ancestry proportions in ESAM were not significantly different than seen in the reference NESAM population (t-test, lowest p = 0.24; **Supplementary Figure S8**), suggesting that differences in effect size and association in our original association were not solely the consequence of mismatched ancestry between ESAM and NESAM. Using TRACTOR to perform logistic regression with 3-way admixture, we found that when conditioned on shared East African ancestry (proxied by LWK), seven loci surpassed the loci-level threshold for association, including candidates *MTHFR1*, *PRICKLE2*, and *PLD2*, but only one OCM locus, and none of our candidates, met the same significance criterion when adjusting for West African ancestry (Fig. 6b-c). We also observed that stratifying our random sampling cumulative association by ancestry background demonstrated a higher cumulative association between variants of presumed East African ancestry compared to West African ancestry at a p-value threshold of 0.001 (z = 2.81 and = 0.54, respectively) (Fig. 6d). Collectively, these findings were consistent with our OCM-ESAM association being driven by variants carried on a shared haplotype background more common among individuals of East African ancestry.

### Signatures of selection at OCM loci among African populations

Periods of famine have been associated with substantial childhood mortality, with survival into adolescence following childhood starvation potentially having a major effect on reproductive fitness. In the absence of modern-day nutritional support, ESAM is associated with higher mortality than NESAM, creating the potential for variants and loci that influence the risk of developing ESAM or NESAM to be subject to relatively strong selection. To explore this further, we generated haplotype-similarity-based scores (iHS) of recent selection at our OCM loci (+/− 50kb) using whole genome sequence data from a subset of 151 individuals from Malawi (Methods). We contextualized our results by considering iHS scores for the same OCM loci generated from genome sequencing done through the H3Africa consortium^65^, which includes samples from Benin, Botswana, Cameroon, Mali, Nigeria, Zambia, and Gur speakers from West Africa (Burkino Faso and Ghana).

**Figure 7:**
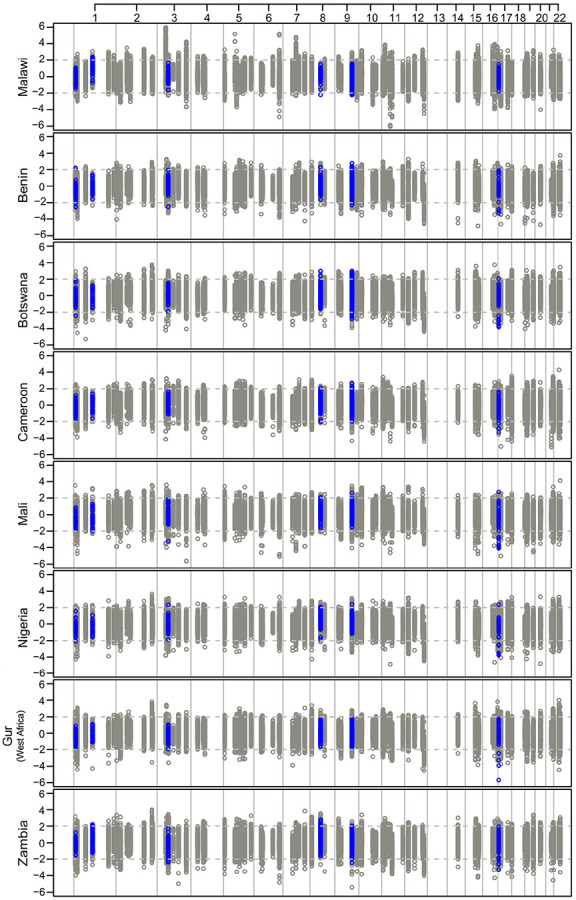
Standardized iHS scores for OCM loci for eight countries/regions in Africa. Grey open circles identify iHS scores within 50kb of OCM genetic loci. Blue circles indicate iHS scores in regions within 10kb of ESAM-associated OCM genetic loci. The dashed lines represent thresholds of +2 and −2 iHS scores, which are suggestive of strong selection. There were no included OCM loci on chromosome 13.

Using an ad hoc, but conservative, locus-selection threshold of more than 10% of SNPs having normalized iHS scores > 2 or <−2, we found that in Malawi, 15 of our 95 OCM loci (15.8%) had some evidence of selection, with *AMT* (0.429), *SDS*/*SDSL* (0.301), *MDH2* (0.208) and *SHMT1* (0.174) having the most SNPs surpassing our threshold (**Supplementary Table S6**). Of these loci, however, none overlapped our top ESAM-associated candidate loci, most of which had proportions < 3%. Across the African countries in the H3Africa dataset, however, 2,440 SNPs within 50kb of OCM genes had evidence of selection in at least two countries (**Supplementary Table S6; Supplementary Figure S9**), with 55 SNPs having strong evidence across all surveyed groups (Fig. 7). In this broader dataset, rs1052748 (ESAM-associated *PLD2* coding variant) had the strongest selection signal among ESAM-associated OCM SNPs (Fig. 7; **Supplementary Table S7**), as did nearby *PLD2* SNPs, particularly in Zambia (proportion = 9.6%), Cameroon (9.3%), and among Gur speakers from West Africa (13.3%).

Lastly, we considered that haplotype similarity scores at selected loci might be amplified among ESAM samples, mimicking the cumulative allelic effects seen in our association models (i.e. enriching for multiple copies of a risk/protective allele). Across all OCM loci, the mean iHS score was significantly higher in ESAM (n = 90) than NESAM individuals (n = 61) (normalized iHS- two sample t-test p = 4x10^−7^; raw iHS - p = 5x10^−14^; **Supplementary Figure S10a**). Among variant sites within 10KB of SNPs surpassing our locus-wide cut-off (n = 839 ESAM; n = 753 NESAM), mean iHS scores were also significantly different (two sample t-test p = 0.01; **Supplementary Figure S10b**) between the two groups.

## DISCUSSION

We report results of a case-control genetic association study of ESAM (affected/cases) versus NESAM (reference/controls) at loci associated with OCM. To our knowledge, this is the largest genetic study of ESAM to date. Our hypothesis-driven targeted pathway approach allowed us to maximize our modest sample size and, in the absence of an available replication cohort, we find strong internal consistency between the two country cohorts assessed. SAM is an acute, life-threatening disorder, for which, traditionally, recruitment, consent, and sampling for large-scale research are secondary considerations; however, the ability to retrospectively recruit individuals several months to years after surviving SAM holds potential for future, larger, germline genetic studies of ESAM. Here, we report evidence of an association between cumulative and locus-specific genetic variation at OCM-loci and the risk of developing ESAM. This association appears to be driven by variants carried on an East African genetic background and is bolstered by evidence that associated variants causally-mediate the association between OCM metabolites and ESAM, as well as by suggestions of recent selection at ESAM-OCM associated loci.

Intronic SNPs in *GABBR2* and *PRICKLE2* showed the strongest locus-specific association. Both loci include multiple sites of reported open chromatin, suggestive of regulatory effects; however, it is unknown whether these effects would be limited to the most proximal genes versus having more distal effects on other *cis-* or *trans-* genes. The latter observation is particularly relevant at *GABBR2* where the top SNP (rs70387285) had a much higher minor allele frequency among African populations. The relative lack of transcriptional or gene-regulatory contexts from Africa makes it difficult to fully annotate the regulatory potential of candidate variants identified in studies such as ours. We did, however, observe locus-wide positive association between a GTEx-reported eQTL missense variant in *PLD2* (rs1052748) and ESAM. The minor allele of rs1052748 is associated with reduced *PLD2* transcription. The functional consequence of variable transcription on phospholipase D2 phosphatidyl activity and production of choline from phosphatidylcholine is uncertain; however, reduced choline availability would be consistent with lower choline concentrations noted in ESAM^31^.

These single locus metabolic effects, however, do not occur in isolation - small changes in metabolic flux at multiple points in OCM could result in larger effects on OCM consistent with those reported in ESAM. This was consistent with both our cumulative association model and our Mendelian Randomization models. For instance, most of the loci reaching locus-wide association were associated with homocysteine metabolism, and MR analyses suggested causal effects of SNPs at *GABBR2, PRICKLE2*, and *PLD2* on metabolites that are either directly derived from homocysteine (Cysteine and Cystathionine) or are involved in its re-conversion to methionine (Betaine). These observations strengthen previous studies implicating aberrant OCM, and particularly deficient re-methylation of homocysteine to methionine, in the pathogenesis of ESAM. These data thus provide support for proposed therapeutic interventions to supplement OCM turnover in SAM using cofactors such as choline (clinicaltrials.gov ID NCT06154174). Despite this, our preliminary estimates suggest whilst genetic variation has a moderately strong effect on the variance in SAM outcomes (estimated heritability = 0.22; standard error (se) = 0.37), variation at OCM loci accounts for a much smaller proportion (estimated heritability = 0.06; se = 0.06). The modest sample size employed means that these heritability estimates have a relatively large standard error as do the effect sizes inferred from our MR analysis. Optimistically, however, there may be several unidentified loci that causally influence ESAM risk. Larger cohorts, particularly from areas where the prevalence of ESAM relative to NESAM remains high, would allow for a well-powered genome-wide approach to identify other ESAM-associated loci and/or pathways for therapeutic and nutritional targeting.

We were able to uniquely leverage African intra-continental admixture patterns to aid in our mapping. The deep ancestral tree within Africa harbors a complex and diverse genetic landscape that can be as disparate as inter-continental variation. The higher rates of ESAM in east-central and southern Africa, therefore, presented an opportunity to employ admixture mapping approaches that have been long proposed for diseases with different population prevalences. We observed evidence of ESAM association at OCM loci when conditioned on East African, but not West African, haplotype backgrounds shared between Malawi and Jamaica. We hypothesized that reducing the ‘noise’ of West African haplotypes in the analysis, would have augmented the effect of shared East African ancestry beyond the effect seen in our primary association. The weaker signal observed likely represents limitations in the representation of African haplotypes in current imputation panels and public databases^81,82^, making imputation and selection of parental ancestral proxies necessarily deficient (e.g. the LWK are an imperfect proxy for the ancestral East African ancestry of Malawi). None the less, our results provide a conceptual model for leveraging admixture within genetically heterogenous groups to genetically map traits with prevalence differences between groups, provided appropriate parental proxies can be identified and there is sufficient genetic distance between the groups in question.

There are several examples of how migration across, into, and out of Africa, has interfaced with varying infectious and non-infectious exposures to shape the African genome through adaptation^83^. More recent studies have implicated multiple novel loci, often with varying effect sizes across the continent^65^. The history of famine and food crises in Africa varies by country and are complex; periods of famine have been documented as far back as the 1600s^84^, with speculation that these were more severe in the 19th century, at least in east and southern African countries such as Zimbabwe and Tanzania^85,86^. At the same time, children developing ESAM have been noted to have higher birth weights^87^, despite the putative link between low birth weight and early-life SAM. These observations provided a starting point for considering that differential survival between ESAM and NESAM might be reflected in signatures of recent selection at ESAM-associated OCM loci. We noted several OCM loci with strong evidence of selection, particularly the serine dehydratase (SDS/SDSH) complex on chromosome 12, which had a strong signal in all the populations evaluated. Among our ESAM-associated OCM loci, however, only *PLD2* showed evidence of selection. Like our genetic association, we observed that OCM selection scores among ESAM individuals were, on average, significantly higher than observed among participants with NESAM, suggesting that we may be similarly underpowered to observe subtle selection signatures at individual loci that contribute to a pathway-wide metabolic effect. Going forward, integration of selection statistics at scale, particularly in the context of African genomic association studies, could provide valuable biological insights, especially for novel loci and understudied disorders.

Genome sequencing and genetic mapping efforts in Africa and among populations of predominantly African ancestry are gaining traction, although the number of reference genomes, particularly for non-West African groups, still vastly under-represents the variation across the continent. Our study provides a starting exemplar for applying a hypothesis-driven, population genetics-informed model to study a disease with high prevalence in specific African population groups. Continued expansion of sequencing efforts in African populations, alongside refinement and deployment of association models that can integrate intra-continental admixture and population genetic statistics, has the potential to maximize the future of human genetics studies on the continent and globally.

## Supplementary Material

This is a list of supplementary files associated with this preprint. Click to download.

• SAMOCMSupplementaryMethodsv2.docx

• SAMOCMSupplementaryTablesv5.xlsx

• SAMOCMSupplementaryfiguresv12.pdf

## Footnotes

WEB RESOURCES H3Africa array v1.1 - https://h3africa.org/index.php/2019/12/12/h3africa-chip-faq/ H3Africa Array manifest - https://www.illumina.com/content/infinium-h3africa-consortium-array-data-sheet) H3ABioNet Imputation Service - https://www.h3abionet.org/resources/h3abionet-imputation-service;h3abionet/chipimputation. Minimac - https://github.com/statgen/Minimac4 University of Cape Town High-Performance Computing - https://ucthpc.uct.ac.za/ 1000 Genomes Project Resource - https://www.internationalgenome.org/ ‘In house scripts’ - https://github.com/NHGRI/SAM_OCMgenetics
